# CCL5-CCR5 interactions modulate metabolic events during tumor onset to promote tumorigenesis

**DOI:** 10.1186/s12885-017-3817-0

**Published:** 2017-12-08

**Authors:** Darrin Gao, Lisa H. Cazares, Eleanor N. Fish

**Affiliations:** 1 Toronto General Hospital Research Institute, University Health Network, Toronto, Canada; 2 Department of Immunology, University of Toronto, Toronto, Canada; 3 Molecular and Translational Sciences Division, U.S. Army Medical Research Institute of Infectious Diseases, Frederick, USA

**Keywords:** Breast cancer, CCL5, CCR5, Glycolysis, Anabolic metabolism

## Abstract

**Background:**

In earlier studies we have shown that CCL5 activation of CCR5 induces the proliferation and survival of breast cancer cells in a mechanistic target of rapamycin (mTOR)-dependent manner and that this is in part due to CCR5-mediated increases in glycolytic metabolism.

**Methods:**

Using the MDA-MB-231 triple negative human breast cancer cell line and mouse mammary tumor virus – polyomavirus middle T-antigen (MMTV-PyMT) mouse primary breast cancer cells, we conducted in vivo tumor transplant experiments to examine the effects of CCL5-CCR5 interactions in the context of regulating tumor metabolism. Additionally, we employed Matrix-Assisted Laser Desorption/Ionization Fourier Transform Ion Cyclotron Resonance Mass Spectrometry imaging (MALDI-FTICR-MSI) to evaluate tumor utilization of cellular metabolites.

**Results:**

We provide evidence that, in the absence of CCR5, the early events associated with rapid tumor growth in the MMTV-PyMT mouse model of spontaneous breast cancer development, are diminished, as demonstrated by a delay in tumor onset. In tumor transplant studies into immunocompromised mice we identify a direct correlation between reduced tumor proliferation and decreased metabolic activity, specifically associated with tumor expression of CCR5. The reduction in tumorigenesis is accompanied by decreases in glucose uptake, glucose transporter-1 (GLUT-1) cell surface expression, intracellular ATP and lactate levels, as well as reduced CCL5 production. Using MALDI-FTICR-MS, we show that the rapid early tumor growth of CCR5^+/+^ triple negative breast cancer cells in vivo is attributable to increased levels of glycolytic intermediates required for anabolic processes, in contrast to the slower growth rate of their corresponding CCR5^−/−^ cells, that exhibit reduced glycolytic metabolism.

**Conclusions:**

These findings suggest that CCL5-CCR5 interactions in the tumor microenvironment modulate metabolic events during tumor onset to promote tumorigenesis.

**Electronic supplementary material:**

The online version of this article (10.1186/s12885-017-3817-0) contains supplementary material, which is available to authorized users.

## Background

Metabolic reprogramming is critical for tumor development [1]. Specifically, during conditions of either nutrient sufficiency or deficiency, tumors will reprogram their metabolic activity towards anabolic or catabolic metabolism, respectively [2–4]. Notably, the Warburg effect of aerobic glycolysis associated with glucose uptake and increases in glycolytic flux enables tumor cells to invoke subsidiary metabolic pathways to support the energetic demands of the proliferating tumor cells [5–7].

The tumor microenvironment is characterized by inflammatory cells and soluble factors that regulate tumor development. Chemokines, including CXCL3, CXCL12, CXCL13, CCL21, CCL2 and CCL5, have been implicated in promoting the malignant phenotype in breast cancer [8–11]. While CCL5 has a role in promoting anti-tumour responses, there is accumulating evidence for CCL5 supporting cancer metastasis and progression [12–14]. Specifically, CCL5 levels are markedly higher in more aggressive forms of breast cancer and are predictive of rapid disease progression in stage II breast cancer patients [15]. Moreover, CCL5 levels are notably more elevated in the sera of patients with high-grade tumors compared to those with low-grade tumors [16]. In addition, CCL5 production by tumors enhances the infiltration of tumor-associated macrophages (TAM) and myeloid derived suppressor cells (MDSCs), leading to the production of growth factors that enhance tumor cell proliferation [14, 17]. TAMs spontaneously produce CCL22, which facilitates the recruitment of regulatory T cells (Tregs) to the tumor microenvironment. The presence of Tregs and MDSCs leads to the production of IL-6 and IL-10 by macrophages, which repress T cell activity thereby resulting in poor anti-tumor immunity [18, 19].

In earlier studies, we showed that CCL5 engagement with its cognate receptor, CCR5, results in the up-regulation of mRNA translation of pro-survival factors leading to enhanced proliferation in MCF-7 breast cancer cells [20]. More recently, we demonstrated that CCL5 activation of CCR5 results in a significant increase in cellular glycolytic activity, specifically increased glucose uptake, GLUT-1 surface expression and increases in intracellular ATP levels, while promoting a global shift towards anabolic metabolism [21]. We provided evidence that CCL5 activation of CCR5 can stimulate proliferation of breast cancer cells in an mTOR-dependent manner.

These data suggest that in addition to a role for CCL5 in the tumor microenvironment influencing the recruitment of immune cells that inhibit anti-tumor immunity, CCL5 also appears to influence tumor metabolism. To interrogate these in vitro findings further, we examined the effects of these CCL5-CCR5 interactions in the context of regulating tumor metabolism, in vivo. We provide evidence that in the absence of CCR5, the early events associated with rapid tumor growth are diminished; our studies identify a direct correlation between reduced tumor proliferation and decreased metabolic activity. The novelty in our finding suggests targeting CCL5-CCR5 interactions may be a potential therapeutic strategy to limit tumor proliferation.

## Methods

### Mice

All mice were housed in a pathogen-free environment and all experiments were approved by the Animal Care Committee of the Toronto General Hospital Research Institute.

C57BL/6 MMTV-PyMT mice were provided by P. Ohashi (University Health Network, Toronto). C57BL/6 CCR5^−/−^ mice were purchased from the Jackson Laboratory. C57BL/6 MMTV-PyMT.CCR5^−/−^ were generated as previously described [21]. Multifocal tumors (100-700 mm^3^) were harvested from MMTV-PyMT.CCR5^+/+^ and MMTV-PyMT.CCR5^−/−^ mice, the tissue minced, then digested with digestion buffer (100 units/ml penicillin, 100 mg/ml streptomycin, 1 mg/ml DNase, 100 U/ml Collagenase type I). The resultant cell suspension was passed through a 40 μm filter to obtain a single cell suspension. The single cell suspensions were maintained in F12 medium supplemented with 10% fetal calf serum (Sigma), 100 units/ml penicillin and 100 mg/ml streptomycin (Invitrogen). NOD scid gamma (NSG) mice were purchased from the Cancer Stemcell Colony at the University Health Network, Toronto. Eight week old female NSG mice were injected with 2.5 × 10^6^ cells in 200 μL PBS into their lower mammary fat pads. Tumor growth was monitored externally using calipers.

### Cells and reagents

Human breast cancer cell line MDA-MB-231 was a gift from L. Penn (University Health Network, Toronto). Cells were maintained in DMEM/F12 medium supplemented with 10% fetal calf serum (Sigma), 100 units/ml penicillin and 100 mg/ml streptomycin (Invitrogen). CCR5 expression was confirmed by flow cytometry using an anti-human (BD BioSciences) and anti-mouse (BioLegend) CCR5 (CD195) antibody. MDA-MB-231.CCR5^−/−^ cells were generated using a commercial clustered regularly interspaced short palindromic repeats/Cas9 (CRISPR/Cas9) CCR5 gene knockout kit (KN216008) from Origene according to the manufacturer’s protocol. GLUT-1 antibodies were obtained from R & D Systems. CCL5 was a generous gift from A. Proudfoot (Geneva Research Centre, Merck Serono International, Switzerland). 2-(N-(7-Nitrobenz-2-oxa-1,3-diazol-4-yl)Amino)-2-Deoxyglucose (2-NBDG), 2-deoxy-D-glucose (2-DG), the ATP bioluminescent assay, the lactate assay kits and Hoechst 33258 were purchased from Sigma. Oligomycin was obtained from Cell Signaling Technology. 3-(4,5-Dimethylthiazol-2-yl)-2,5-Diphenyltetrazolium Bromide (MTT) and puromycin were obtained from ThermoFisher Scientific. Anti-mouse CD31, CD45 antibodies and DAPI were purchased from eBioscience.

### Fluorescence-activated cell sorting (FACS) analysis

10^6^ cells were incubated with the specified primary antibodies, or isotype control antibodies, for 30 min at room temperature, followed by 30 min with FITC/PE-conjugated secondary antibodies. Cells were analyzed using the FACSCalibur and FlowJo software (BD Biosciences).

### Glucose uptake assay

3 × 10^5^ cells in 2% FCS DMEM/F12 medium were plated in individual wells of 12-well plates overnight. Cells were either left untreated, treated with inhibitors or treated with CCL5 alone for the indicated times, then pulsed with 50 μM 2-NBDG for 45 min. Cells were washed three times with PBS, detached using 1 mM EDTA, and analyzed using the FACSCalibur and FlowJo software (BD Biosciences).

### ATP bioluminescent assay

Intracellular ATP levels were examined using the ATP bioluminescent assay kit, according to the manufacturer’s protocol (Sigma). Briefly, 2 × 10^4^ cells were either left untreated, treated with inhibitors or treated with CCL5 alone. Bioluminescence was measured using a VICTOR™ X3 Multilabel Plate Reader (PerkinElmer).

### Lactate assay

Intracellular lactate levels were examined using the lactate assay kit, according to the manufacturer’s protocol (Sigma). 2.5 × 10^5^ cells were either left untreated, treated with inhibitors for 1 h or treated with CCL5. 0.1 g of tissue was used for all ex vivo lactate assays. Fluorescence readings were measured using a VICTOR™ X3 Multilabel Plate Reader (PerkinElmer).

### MTT proliferation assay

1 × 10^4^ cells were seeded into individual wells of 96-well plates in 2% FCS DMEM/F12. Cells were either left untreated, treated with inhibitor or CCL5 for 4 days. On day 4, 10uL of 12 mM MTT stock solution was added to each well and incubated for 4 h. 50uL of of DMSO was added to each well before measuring absorbance.

### ELISA

CCL5 levels in culture supernatants were measured using anti-mouse CCL5 (DY478) and anti-human CCL5 (DRN00B) ELISA kits from R&D Systems, according to the manufacturer’s protocol.

### Hemtaoxylin & eosin staining of thin sections

Mice were euthanized, their tumors harvested, then fixed in 10% formalin. Thin sections (5 μm) were prepared and stained with hematoxylin and eosin (H & E).

### Immunofluorescence staining

Tumors were harvested and immediately frozen in liquid nitrogen. 8 μm thin sections were cut. Thin sections were fixed with 10% formalin and blocked with 1%BSA, then stained for immune infiltrates [CD45-FITC] and endothelial cells [CD31-PE]. DAPI (4′6-diamidino-2-phenylindole) staining was included to identify nuclei (A-T DNA stain). Sections were visualized using a Zeiss LSM700 Confocal microscope and quantitated using Aperio ImageScope (V12.3).

### [^18^F]FDG glucose uptake and tumor perfusion in vivo

10 MBq of [^18^F]FDG was injected intravenously (iv) into mice for 1 h. Live mice were then scanned using a microPET scanner (Siemens Focus 220) to detect fluorescent uptake. Mice were also scanned with a microCT scanner (GE Locus Ultra) to collect anatomical information to assist with tumor volume quantification. One minute prior to being euthanized, mice were injected iv with 40mgkg^−1^ of Hoechst 33,258. Tumors were harvested, frozen in liquid nitrogen and sectioned at 8 μm. Tumor perfusion was measured using ImageJ after visualizing the sections with UV illumination. Specifically, the Hoeschst dye was UV activated at 350 nm, then absorbance measured at 460 nm.

### Matrix-Assisted Laser Desorption/Ionization Fourier Transform Ion Cyclotron Resonance Mass Spectrometry (MALDI-FTICR-MS) imaging

#### Materials

9-Aminoacridine hydrochloride (9-AA) was purchased from Sigma–Aldrich. Water and Methanol was purchased from Fischer Scientific at the highest purity grade available and used without further purification.

#### Tissue preparation for MALDI-FTICR-MS imaging

Frozen tumor tissue sections (10 μm) were prepared with a Microm HM550 (Thermo Scientific) cryostat and were placed on histology slides for MALDI-FTICR-MS imaging. Serial sections (5 μm) were hematoxylin and eosin (H&E) stained for morphological analysis. Imaging slides were stored at −20 °C and were then slowly brought to room temperature in a desiccator and prepared for MALDI-MSI analysis. A uniform coating of 9-AA was added using an HTX TM Sprayer (HTX Technologies Carrboro, NC, Germany) and a solution of 10 mg/mL 9-AA in 80:20 MeOH/H_2_O. Slides were then stored in a desiccator for 1 h before analysis in the mass spectrometer.

#### MALDI-FTICR-MS imaging data acquisition

All experiments were performed using a 7 T solariX XR FTICR mass spectrometer (Bruker Daltonics, Billerica, MA, USA) using a 50-μm pixel size and 200 laser shots per pixel. The FTICR mass spectrometer was calibrated prior to the MALDI-MSI experiments using the peaks of NaTFA. Each pixel’s mass spectrum was then recalibrated using the matrix peak of 9-AA as an internal lock mass during data collection. Mass spectra were acquired in negative ion mode within a mass range of 150–1100 m/z and 1 M data points collected. Molecular images were visualized using the FlexImaging software (version 4.1, Bruker) using total ion current normalization for visualization of images.

#### Discriminate metabolite analysis

Data from FlexImaging was exported to SCiLS Lab (ver.2015b-3.02.7804: Bremen, Germany) was used for processing the datasets as follows: Raw data was imported and normalized to total ion current (TIC). To determine peaks with differential abundance in WT and KO tumor tissue, 200 random spectra from each region were selected for discriminate analysis in SCiLS. Hypothesis testing was then performed using the Student’s t-test on the mean spectra of each tissue region for each m/z region identified. To obtain the most accurate m/z for potential metabolite peaks, data from FlexImaging were exported into Data Analysis (version 4.4 Bruker) and internal calibration was performed using 9AA, ATP and ADP (endogenous tissue metabolite peaks, verified using an ATP standard). Compounds were identified by accurate mass match of obtained high resolution FTICR m/z values to the Metlin database of metabolites. High mass accuracy, using a mass tolerance of 1 ppm, was then used to filter the discriminate peaks and assign provisional identifications.

### Statistical analyses

Statistical significance was analyzed by Student’s t-test unless otherwise specified. A level of *p < 0.05* identified significance. Data are expressed as mean ± SE (standard error of means).

## Results

**Fig. 1:**
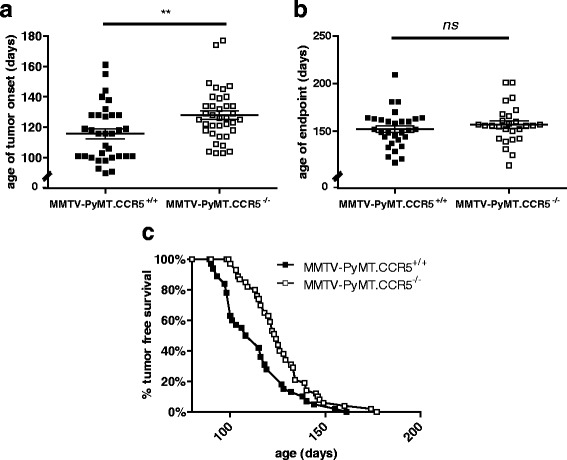
MMTV-PyMT.CCR5^−/−^ mice have delayed tumor onset and increased tumor free survival compared to MMTV-PyMT.CCR5^+/+^ mice. **a**, **b** MMTV-PyMT.CCR5^+/+^ and MMTV-PyMT.CCR5^−/−^ mice were monitored daily for palpable tumors, using calipers. Tumor onset is defined as detection of a palpable tumor, volume ~20mm^3^. Tumor endpoint is defined as a palpable tumor with volume ~700mm^3^. Mice were euthanized when tumors reached this size. Each data point represents a single mouse. Means and SEM are identified as horizontal lines. **c** Tumor-free survival rate is the percentage of the mice in the colony that are tumor-free at each specified age. * * p < 0.01

In a first series of experiments we employed the MMTV-PyMT mouse model to understand the role of CCL5-CCR5 in modulating tumor metabolism in vivo. MMTV-PyMT mice express the polyomavirus middle T-antigen under the control of mouse mammary tumor virus LTR [22]. PyMT is a potent oncogene that encodes a transmembrane protein which activates several signal transduction pathways, including those of the *Src and ras* family and PI3 kinase pathways [23]. PyMT expression is also associated with increased c*-myc* levels, leading to prolonged cell survival [23]. Female MMTV-PyMT mice develop mammary hyperplasia and exhibit an average onset of palpable tumors by 15 weeks of age. To examine the effects of CCL5-CCR5 activation on breast cancer development, we generated MMTV-PyMT.CCR5^−/−^ mice by breeding MMTV-PyMT.CCR5^+/+^ mice with C57Bl/6.CCR5^−/−^ mice, as described earlier [21]. In a first series of experiments we identified a delay of 12.1 days in palpable tumor detection (~20mm^3^), hereafter defined as tumor onset, in the MMTV-PyMT.CCR5^−/−^ mice compared with the MMTV-PyMT.CCR5^+/+^ mice (Fig. 1a). Further, we observed a 4.8-day delay in tumour endpoint, hereafter defined as ~700mm^3^ (Fig. 1b). Notably, the MMTV-PyMT.CCR5^−/−^ mice exhibited an increase in tumour-free survival rate (Fig. 1c). In situ CT examination of tumors in live mice revealed that tumor size was smaller for the MMTV-PyMT.CCR5^−/−^ mice compared with the MMTV-Py.MT.CCR5^+/+^ mice (Additional file 1: Figure S1, panel B). Moreover, using [^18^F]FDG glucose as a measure of in vivo glucose uptake by these tumors and microPET scanner imaging, the data revealed that glucose uptake was reduced in the MMTV-PyMT.CCR5^−/−^ tumors, compared with the MMTV-PyMT.CCR5^+/+^ tumors, when tumors of similar size were compared (Additional file 1: Figure S1, panel C). In situ Hoescht staining of tumors in the live mice, as an indicator of perfusion, identified that the perfusion index for the MMTV-PyMT.CCR5^−/−^ tumors was significantly lower than that for the MMTV-PyMT.CCR5^+/+^ tumors (Additional file 2: Figure S2). When examined for immune cell infiltrates, 0.4-0.5 cm diameter tumors harvested from 3 MMTV-PyMT.CCR5^+/+^ and 3 MMTV-PyMT.CCR5^−/−^ mice 18 days post-tumor onset exhibited no significant differences in CD45+ leukocyte infiltrates; MMTV-PyMT.CCR5^+/+^ derived tumors: CD45 staining index (signal intensity per field normalized over background) = 181 ± SEM 11 per field (20× magnification), 3 fields per thin section. MMTV-PyMT.CCR5^−/−^ derived tumors: CD45 staining index = 166 ± SEM 10 per field (Additional file 3: Figure S3).

Leukocytes are present in the tumor microenvironment, including tumor-infiltrating lymphocytes, innate lymphoid cells, tumor-associated macrophages and myeloid-derived suppressor cells. CCR5 is important for the recruitment of immune cells to the tumor microenvironment and CCL5 for their secretion of pro-tumorigenic factors [24]. In order to reduce the effects of CCL5-CCR5 mediated immune cell recruitment to the tumor microenvironment that would influence tumor proliferation, we harvested tumors from MMTV-PyMT.CCR5^+/+^ and MMTV-PyMT.CCR5^−/−^ mice, prepared tumor cell suspensions, then injected these cell suspensions into mammary fat pads of NSG mice, as described in the Methods section. NSG mice lack T, B and NK cells and have defective antigen presenting cells and functionally immature macrophages [25]. This experimental strategy allows for the investigation of the direct effects of CCL5-CCR5 activation on the tumor cells, specifically in our context of metabolic activation.

**Fig. 2:**
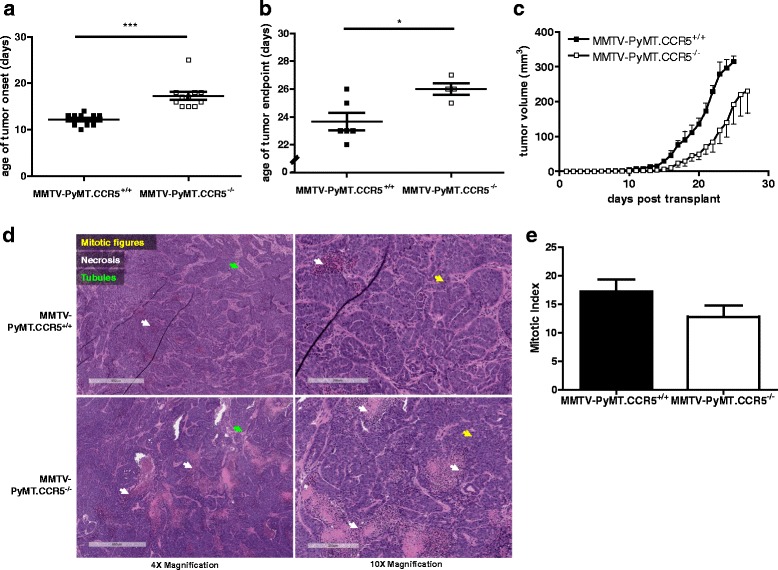
MMTV-PyMT.CCR5^−/−^ tumor cells transplanted into NSG mice exhibit delayed tumor growth compared with MMTV-PyMT.CCR5^+/+^ tumor cells. Tumors (100-700 mm^3^) from MMTV-PyMT.CCR5^+/+^ and MMTV-PyMT.CCR5^−/−^ mice were harvested and injected into the lower mammary fat pads of NSG recipients (*n* = 12), as described in Methods. Tumor growth was monitored externally using calipers. **a** Time to palpable tumor onset (~20mm^3^) and **b** tumor endpoint (~700mm^3^). Each data point represents a single mouse. **c** Tumor volume was measured over time to determine the rate of tumor growth. Means and SEM are identified as horizontal lines. **d** Onset tumors were harvested, fixed, thin sections prepared and stained with H & E. Arrows show indicated features. **e** The average number of mitotic figures in each of 5 fields/thin section at 20× magnification are recorded. * p < 0.05 and *** p < 0.001

In a first series of experiments we observed a delay in the onset of palpable tumors (~20mm^3^) when MMTV-PyMT.CCR5^−/−^ cells are transplanted into NSG mice compared with transplanted MMTV-PyMT.CCR5^+/+^ cells, of the order of 4.9 days (Fig. 2a). Moreover, we consistently observed a delay of >2 days to tumor endpoint (~700mm^3^) for the MMTV-PyMT.CCR5^−/−^ tumors compared with the MMTV-PyMT.CCR5^+/+^ tumors (Fig. 2b). Furthermore, tumor volume remained lower when MMTV-PyMT.CCR5^−/−^ cells were transplanted compared with MMTV-PyMT.CCR5^+/+^ cells (Fig. 2c). Onset tumors were harvested, fixed in 10% formalin, thin sections prepared and stained with H&E. Both MMTV-PyMT.CCR5^+/+^ and MMTV-PyMT.CCR5^−/−^ tumors were identified as high grade adenocarcinomas with nuclear pleomorphism, high mitotic count (mitotic index for MMTV-PyMT.CCR5^+/+^ tumors = 17 ± SEM 2; mitotic index for MMTV-PyMT.CCR5^−/−^ tumors = 13 ± SEM 2) and low tubule formation (Fig. 2d, e). Adenocarcinomas often form glandular structures. Typically, a lack of tubules signifies a more advanced grade of cancer. In addition, vesicular nuclei, often large and irregularly shaped, and high mitosis rates are classical hallmarks of high grade tumors [26].

**Fig. 3:**
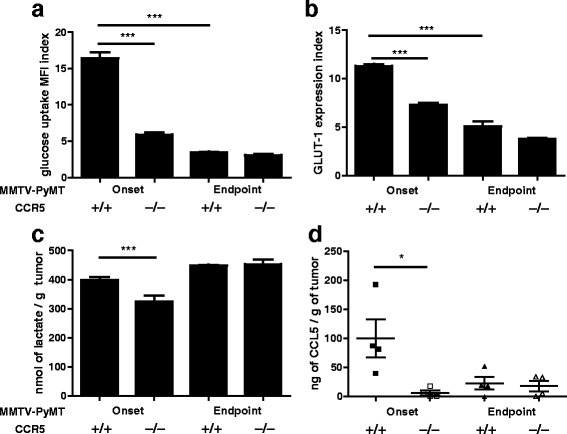
MMTV-PyMT.CCR5^−/−^ tumor cells harvested from NSG mice exhibit lower glucose uptake, reduced GLUT-1 expression and lower levels of cellular metabolites than MMTV-PyMT.CCR5^+/+^ tumor cells. MMTV-PyMT.CCR5^+/+^ and MMTV-PyMT.CCR5^−/−^ tumors were harvested from recipient NSG mice when they were either just palpable (~20mm^3^), i.e. tumor onset, or ~700mm^3^, endpoint. Following 16 h in culture, **a** glucose uptake, **b** GLUT-1 expression, **c** intracellular lactate and **d** CCL5 production, were measured. Values are the means +/− SEM of technical triplicates. *p < 0.05, ** p < 0.01, *** p < 0.001

We next compared MMTV-PyMT.CCR5^+/+^ and MMTV-PyMT.CCR5^−/−^ tumour cells by examining their metabolic status ex vivo. We found that both MMTV-PyMT.CCR5^+/+^ and MMTV-PyMT.CCR5^−/−^ tumors were more metabolically active during onset (~20mm^3^) compared to endpoint (~700mm^3−^), as demonstrated by higher glucose uptake (Fig. 3a) and surface GLUT-1 expression (Fig. 3b). Notably, at onset, MMTV-PyMT.CCR5^+/+^ tumours were more metabolically active than MMTV-PyMT.CCR5^−/−^ tumors, given the same tumor volume. MMTV-PyMT.CCR5^+/+^ tumors exhibited higher cellular lactate levels than MMTV-PyMT.CCR5^−/−^ tumors at onset, potentially due to a higher rate of glycolysis (Fig. 3c). However, by endpoint, lactate levels have increased and no differences were identified between MMTV-PyMT.CCR5^+/+^ and CCR5^−/−^ tumor cells. MMTV-PyMT.CCR5^+/+^ tumors also produced the highest levels of CCL5 ex vivo when harvested at both onset and endpoint, with consistently higher CCL5 levels detectable at onset compared with endpoint (Fig. 3d).

**Fig. 4:**
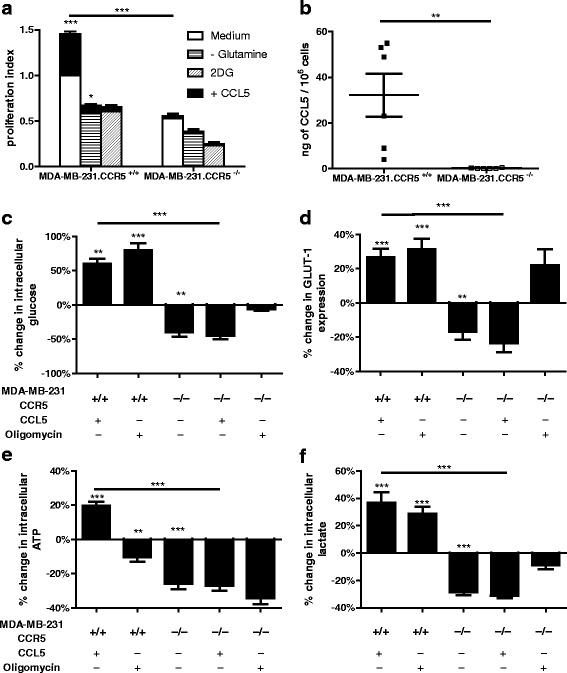
In vitro, MDA-MB-231.CCR5^−/−^ cells are less metabolically active than MDA-MB-231.CCR5^+/+^ cells. **a** Cells were either left untreated (medium alone), treated with 10 nM CCL5, or pre-treated with 2 mM 2-DG for 1 h prior to CCL5 treatment, or maintained in medium containing 5 mM glutamine. For all, medium was changed every other day and the treatment(s) reapplied. Cell proliferation was quantified using an MTT assay as described in Methods. The proliferation index is normalized against untreated conditions (ie medium alone). Values are means ± SEM of triplicate assays and each data point combines the data from 3 independent experiments. Statistical analysis was performed comparing untreated cells with CCL5-treated cells and inhibitor-treated cells with CCL5 + inhibitor treated cells, or comparing CCL5-treated with CCL5 + inhibitor-treated cells. **b** CCL5 levels were measured from culture supernatants after 16 h incubation. **c** Glucose uptake, **d** GLUT-1 expression, **e** intracellular ATP and **f** intracellular lactate were measured as described in Methods in cells treated with 10 nM CCL5 or 2 μM oligomycin for 3 h. Data are expressed as percent-change relative to untreated MDA-MB-231.CCR5^+/+^ cells. Values are means ± SEM of triplicate assays and each data point combines the data from 3 independent experiments. * p < 0.05, ** p < 0.01, *** p < 0.001

One of the most aggressive and malignant types of breast cancer is the triple negative (estrogen receptor- (ER-), progesterone receptor- (PR-), human epidermal growth factor receptor 2- (HER2-)) basal-like adenocarcinoma. MDA-MB-231 is a widely used triple negative human breast cancer cell line that expresses CCR5 [21, 27]. Accordingly, to further interrogate the contribution of CCR5 to breast cancer metabolism, we generated MDA-MB-231.CCR5^−/−^ tumor cells using CRISPR/Cas9 technology (Additional file 4: Figure S4). In an earlier publication we provided evidence that CCL5 treatment enhances the proliferation of MDA-MB-231.CCR5^+/+^ cells, which is dependent on glucose and glutamine metabolism [21]. The MDA-MB-231.CCR5^−/−^ cells exhibit a lower basal proliferation rate compared to MDA-MB-231.CCR5^+/+^ and their growth rate is unaffected by CCL5 treatment (Fig. 4a). Specifically, in medium deficient in glutamine and in medium containing 2-DG, CCL5-inducible increased cell proliferation is diminished in MDA-MB-231.CCR5^+/+^ cells and absent in MDA-MB-231.CCR5^−/−^ cells. In addition, MDA-MB-231.CCR5^+/+^ cells secrete CCL5 at physiological levels [27, 28], while CCL5 secretion was undetectable from the MDA-MB-231.CCR5^−/−^ cells, likely due to a lack of CCL5-CCR5 autocrine signaling (Fig. 4b). MDA-MB-231.CCR5^+/+^ and CCR5^−/−^ cells were further analyzed for their rates of glucose uptake (Fig. 4c), surface GLUT-1 expression (Fig. 4d), intracellular ATP (Fig. 4e) and lactate levels (Fig. 4f). MDA-MB-231.CCR5^−/−^ cells exhibited a lower basal rate of metabolism, as evidenced by their lower glucose uptake, GLUT-1 expression and intracellular ATP and lactate levels. As anticipated, MDA-MB-231.CCR5^−/−^ cells were not responsive to CCL5 treatment.

**Fig. 5:**
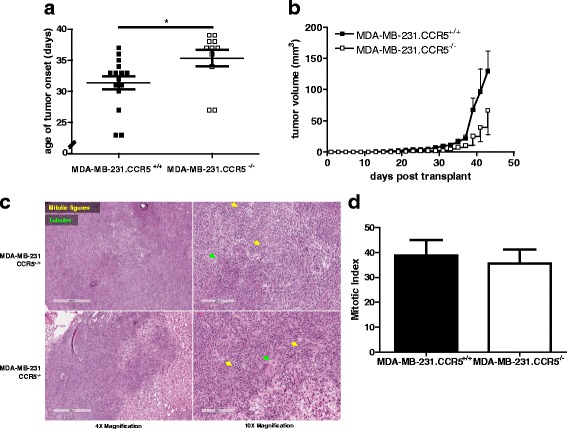
MDA-MB-231.CCR5^−/−^ cells transplanted in NSG mice exhibit delayed tumor growth compared with MDA-MB-231.CCR5^+/+^ cells. MDA-MB-231.CCR5^−/−^ and MDA-MB-231.CCR5^+/+^ cells were injected into mammary fat pads of NSG mice (n = 12), as described in Methods. Tumor growth was monitored externally using calipers. **a** Time to palpable tumor onset (~20mm^3^). Each data point represents a single mouse. **b** Tumor volume was measured over time to determine tumor growth. Means and SEM are identified as horizontal lines. **c** Onset tumors were frozen, thin sections prepared and stained with H & E. Arrows show indicated features. **d** The average number of mitotic figures in each of 5 fields/thin section at 20× magnification are recorded.* p < 0.05

**Fig. 6:**
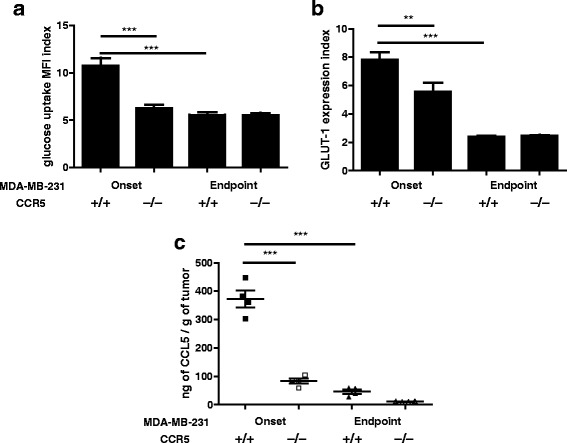
MDA-MB-231.CCR5^−/−^ tumor cells harvested from NSG mice exhibit lower glucose uptake, reduced GLUT-1 expression and lower levels of cellular metabolites than MDA-MB-231.CCR5^+/+^ tumor cells. MDA-MB-231.CCR5^+/+^ and MDA-MB-231.CCR5^−/−^ tumors were harvested from recipient NSG mice when they were either just palpable (~20mm^3^), i.e. tumor onset, or ~700mm^3^, endpoint. Following 16 h in culture, **a** glucose uptake, **b** GLUT-1 expression, and **c** CCL5 production, were measured. Values are the means ± SEM of technical triplicates. ** p < 0.01 and *** p < 0.001

We next examined the proliferative capacity of the MDA-MB-231.CCR5^+/+^ and MDA-MB-231.CCR5^−/−^ tumor cells in vivo. As above, we employed NSG mice, to eliminate/dramatically diminish the contributions of CCL5-CCR5 interactions on an immune response that would influence tumor growth. MDA-MB-231.CCR5^+/+^ and MDA-MB-231.CCR5^−/−^ cells were transplanted into the fat pads of NSG recipient mice. Similar to the MMTV-PyMT transplant study, NSG mice transplanted with MDA-MB-231.CCR5^−/−^ cells exhibited a lower tumor load (Fig. 5a) and on average a 3.96 day delay in tumor onset (Fig. 5b). H&E staining of tumor thin sections revealed that both MDA-MB-231.CCR5^+/+^ and MDA-MB-231.CCR5^−/−^ tumors were high grade adenocarcinomas with nuclear pleomorphism, high mitotic count (mitotic index for MDA-MB-231-CCR5^+/+^ tumors = 9 ± SEM 2; mitotic index for MDA-MB-231.CCR5^−/−^ tumors = 6 ± SEM 6) and low tubule formation (Fig. 5c, d).When tumors were harvested at onset, then cultured ex vivo, MDA-MB-231.CCR5^+/+^ cells had a higher rate of glucose uptake and GLUT-1 expression compared to MDA-MB-231.CCR5^−/−^ cells. When tumors were harvested at endpoint, glucose metabolism was significantly lower and no differences were observed between MDA-MB-231.CCR5^+/+^ and MDA-MB-231.CCR5^−/−^ cells (Fig. 6a, b). The levels of CCL5 produced were significantly higher (approximately 4-fold) from MDA-MB-231.CCR5^+/+^ compared with MDA-MB-231.CCR5^−/−^ cells, when tumors were harvested at onset, yet this production diminished significantly at endpoint, albeit lower CCL5 levels were produced by MDA-MB-231.CCR5^−/−^ compared with MDA-MB-231.CCR5^+/+^ cells (Fig. 6c).

**Fig. 7:**
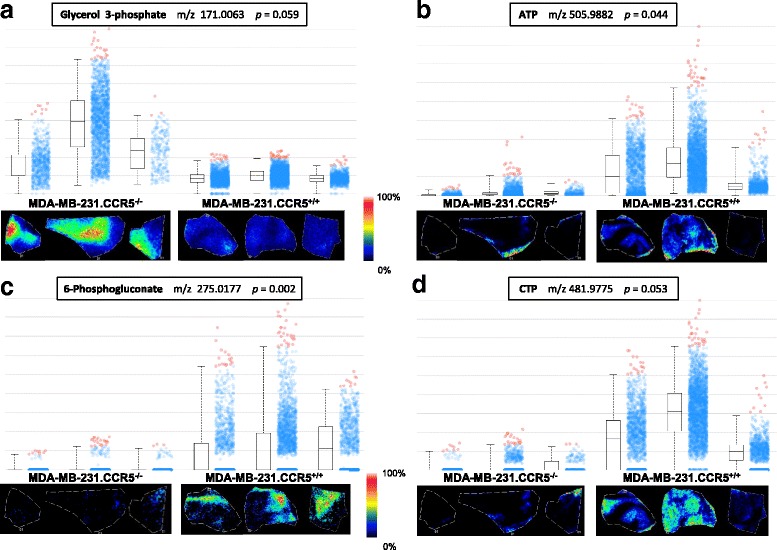
MALDI-FTICR-MSI analysis confirms that MDA-MB-231.CCR5^−/−^ cells are less metabolically active than MDA-MB-231.CCR5^+/+^ cells. Tumor tissue was harvested at tumor onset from NSG mice that had received MDA-MB-231.CCR5^−/−^ or MDA-MB-231.CCR5^+/+^ tumor cell suspensions, rapidly frozen, then sectioned in a cryostat, sprayed with 9AA matrix and subjected to MSI using MALDI-FTICR and a raster width of 50 μm over a m/z range of 150-1100. Each panel (**a**-**d**) represents an identified metabolite. Each box-plot contains a rectangle divided by a horizontal line, which represents the median intensity of that metabolite. Lower and upper bounds of the box represent the second and third quartile. Lines extending vertically from the box represent lower and upper quartiles (0% and 99% respectively). The cloud part of the plot shows how spectra from a given region are spread by intensity for each metabolite and region. Blue dots represent the spectra in-which intensities are between the lower and upper quartiles. Red dots represent outliers. Images below the box plots are show the relative abundance of each metabolite for each tissue (normalized to TIC) and based on the intensity scale provided

To further explore the metabolic differences in MDA-MB-231.CCR5^−/−^ and MDA-MB-231.CCR5^+/+^ tumors, MALDI-FTICR-MS imaging was performed. MS imaging provides the in-situ distribution profiles of metabolites within tissues and FTICR-MS provides the highest level of mass resolution necessary for identification of low molecular weight metabolites [29]. A broad spectrum of analytes ranging from proteins, peptides, small molecule drugs and their metabolites, as well as endogenous cell metabolites, and lipids are identifiable by this technology [30]. Following transfer of MDA-MB-231.CCR5^−/−^ and MDA-MB-231.CCR5^+/+^ tumors into the mammary fat pads of NSG mice and a growth period of 23-40 days, intact tumors were harvested and immediately frozen in liquid nitrogen. Frozen thin sections were mounted onto slides and coated uniformly with a matrix solution of 9-AA (10 mg/ml in 70% MeOH). This matrix formulation ensures robust efficiency for the ionization of important cellular metabolites in tissue samples [31]. Serial sections were stained with H&E and examined to ensure absence of necrotic cells. FTICR-MS data were collected and metabolite ions identified using a metabolite database [32]. Only those peaks detected within 1 ppm of the expected m/z of the [M–H] − metabolite ions were assigned metabolite identities. Metabolite images displaying the relative abundance and box/cloud-plots depicting normalized peak intensities for metabolites identified from 3 MDA-MB-231.CCR5^−/−^ and 3 MDA-MB-231.CCR5^+/+^ transplanted tumor tissues are shown in Fig. 7. ATP is significantly reduced (*p* = 0.044) in the MDA-MB-231.CCR5^−/−^ tumors, and CTP reduction approaches significance (*p* = 0.053). We also observed a marked reduction in levels of 6-phosphogluconate in the MDA-MB-231.CCR5^−/−^ tumors. Conversely, glycerol phosphate, a molecule reduced from DHAP, is increased in abundance in MDA-MB-231.CCR5^−/−^ tumors.

## Discussion

The metabolic phenotype of cells in the tumor microenvironment is heterogeneous, wherein the majority of cells mobilize glucose by glycolysis and others via mitochondrial oxidative phosphorylation [33]. Glycolysis and mitochondrial metabolism are important both for ATP generation and for the biosynthesis of cellular components. Indeed, given the aggressive growth requirements of tumor cells, they exhibit higher metabolic activity rates compared with normal cells; tumour cells metabolize glucose, glutamine and other nutrients at much higher rates than differentiated cells. Notably, tumor cells predominantly utilize aerobic glycolysis over oxidative phosphorylation [34]. While glycolysis generates 2 mol of ATP per mole of glucose, the complete oxidation of glucose through the electron transport chain produces 18 times more. Despite being the less efficient form of oxidative breakdown of glucose, glycolysis will produce ATP 100 times faster than oxidative phosphorylation, while also generating key metabolic intermediates for anabolic processes, mediated by activating the pentose phosphate pathway and amino acid biosynthesis [35].

Onset of tumorigenesis is a critical period when a tumor-supportive microenvironment is established, often facilitated by an unresolved inflammatory response [36]. According to a recent meta-analysis, tumour onset can be directly attributed to infection and inflammation in approximately 15% of cancers [37, 38]. Various cell types accumulate during onset of tumorigenesis, including stromal cells, which contribute to the development of a pro-tumorigenic niche [39]. Abnormal stromal cells can directly induce tumor promotion [12, 40]. Stromal cells produce enzymes, growth factors and cytokines to enable tumor growth [41, 42]. Fibroblasts, the most common types of stromal cells found in the tumor microenvironment, actively participate in tumorigenesis and are responsible for the production of many factors, including extracellular matrix proteins and matrix metalloproteinases [42, 43]. Once the tumor microenvironment is established, cancer-associated fibroblasts (CAFs) become distinctly identifiable. These CAFs induce additional alterations in tumor cells that allow them to metastasize more efficiently [43]. Stromal cells, along with mesenchymal stem cells, are the major sources of chemokines, including CCL5, in the tumor microenvironment. It is now clear that chemokines exert important roles in regulating inflammation, proliferation, survival and migration of tumor cells during the neoplastic process [21, 44]. CCL5 and CCR5 (but not CCR1 and CCR3) are overexpressed in the basal and HER-2+ breast cancer subtypes [27]. The oncogenic transformation of human breast epithelial cells is accompanied by an upregulation in the expression of CCR5, further supporting a role for CCL5-CCR5 interactions in the regulation of tumor development [27, 45]. CCL5 has a crucial role in the metastasis of breast cancer cells [46] and expression of CCR5 on breast cancer cells enhances cellular invasion by 40-fold [47].

Immune cell infiltration is vital for establishing the tumor microenvironment [48]. Recruitment of inflammatory cells to tumors can facilitate disease progression and promote metastasis. CCL5 has been implicated in the recruitment of CD4 + CD25 + Foxp3+ Tregs, leading to the generation of a suppressive environment that supports tumor tolerance [49]. MDSCs, a cell population with immune-suppressive properties, secrete large amounts of CCL5, further promoting the recruitment of Tregs [50, 51]. Additionally, CCR5 expressing- TAM [44] accumulation occurs in hypoxic regions in growing tumors and their accumulation correlates with angiogenesis, a prerequisite to the subsequent invasive phenotype of carcinoma [36]. The tumor microenvironment is more acidified at endpoint, presumably due to an accumulation of lactate, which can stabilize hypoxia-inducible factor 1-alpha (HIF-1α) and leads to vascular endothelial growth factor (VEGF) expression, triggering angiogenesis and invasion [52]. Clearly, CCL5-CCR5 activation in the tumor microenvironment is critical.

Employing the MMTV-PyMT mouse model of spontaneous breast tumor development, we generated MMTV-PyMT.CCR5^−/−^ mice and identified a 12 day delay in tumor onset in these mice compared to the MMTV-PyMT.CCR5^+/+^ mice, yet no discernible differences in tumor endpoint. These findings are consistent with the notion that the pro-survival and metabolic benefits of CCL5-CCR5 signaling are more critical during the onset of tumorigenesis, prior to the formation of a fully established tumor microenvironment, compared to tumor endpoint, when the lack of CCL5-CCR5 signaling may be compensated by other factors as a result of a fully acidified and hypoxic tumor [53–55]. Moreover, we provide evidence that there is less glucose uptake by MMTV-PyMT.CCR5^−/−^ tumors, in vivo, compared with MMTV-PyMT.CCR5^+/+^ tumors, reflected in reduced tumor volume. When tumors from MMTV-PyMT.CCR5^+/+^ and MMTV-PyMT.CCR5^−/−^ mice were transplanted into NSG mice, we likewise observed a delay in tumor onset in recipient mice that received MMTV-PyMT.CCR5^−/−^ cells. The delay in tumor onset employing this model was approximately 4 days, compared with the 12 days in the spontaneous MMTV-PyMT mouse model, likely reflective of the more aggressive progression of tumorigenesis in the immunocompromised NSG mice. Importantly, our data confirm that CCR5 expression on tumor cells directly contributes to tumor proliferation.

Our studies with the triple negative MDA-MB-231.CCR5^−/−^ cells revealed that in the absence of CCR5 expression MDA-MB-231cells do not secrete and are unable to respond to CCL5, have lower glucose uptake and GLUT-1 expression, and lower intracellular ATP and lactate levels. Indeed, the MDA-MB-231.CCR5^−/−^ cells are more sensitive to inhibitors blocking glucose uptake and glutamine catabolism and have a lower proliferation rate than MDA-MB-231.CCR5^+/+^ cells, in further support of a role for CCL5 activation of CCR5 contributing to tumor cell metabolism. Comparing the metabolic status of MDA-MB-231.CCR5^+/+^ and MDA-MB-231.CCR5^−/−^ tumor cells during onset and endpoint we provide evidence that cells are more metabolically active during the onset of tumorigenesis, characterized by lower GLUT-1 expression and lower glucose uptake.

MALDI-FTICR MSI analysis revealed an increase in 6-phosphogluconate, CTP and ATP levels in MDA-MB-231.CCR5^+/+^ tumors compared to MDA-MB-231.CCR5^−/−^ tumors. 6-phosphogluconate is a key intermediate in the pentose phosphate pathway and is converted to ribulose 5-phsophate by phosphogluconate dehydrogenase catalysis. Accumulation of 6-phosphogluconate enables the shuttling of intermediates into anabolic pathways for macromolecule biosynthesis, a metabolic signature that supports cell proliferation and invasion. In addition to serving as high energy molecules, ATP and CTP are also involved with RNA and glycerophospholipid synthesis. High ATP levels further serve to antagonize the AMP-activated protein kinase (AMPK) while promoting the activation of mTOR and energy-intensive protein biosynthesis, another hallmark of anabolic metabolism. MDA-MB-231.CCR5^−/−^ tumors had higher levels of glycerol 3-phosphate. Conversion of dihydroxyacetone phosphate to glycerol 3-phosphate – the glycerol 3-phosphate shuttle – is a mechanism whereby NADH that accumulates in the cytosol during glycolysis may be utilized to generate ATP by mitochondrial oxidative phosphorylation, thereby coupling glycolysis and mitochondrial ATP production. Given the slower rate of glycolysis in MDA-MB-231.CCR5^−/−^ tumors, we infer that the increase in glycerol 3-phosphate levels in the MDA-MB-231.CCR5^−/−^ cells could be a compensatory mechanism to increase ATP production through oxidative phosphorylation by generating additional high potential equivalents via the glycerol 3-phosphate shuttle.

## Conclusion

Viewed altogether, the data indicate that CCL5 activation of CCR5 in breast cancer cells is associated with increased metabolic activity during tumor onset. This increase in glucose uptake and ATP generation fuels the energy and biosynthetic demands of tumor cells, enhancing cell proliferation and tumorigenesis. Our findings are consistent with published studies that have identified the importance of CCR5 in breast tumor progression [27, 56]. Our results suggest that, even in the absence of immune cells, the expression of CCR5 on tumor cells enables tumor growth in an environment where CCL5 is produced, mediated by upreguation of metabolic events. Our ongoing studies are directed towards characterizing the significance of the effects of CCL5-CCR5 interactions on increasing the metabolic activity of tumor cells, specifically in the context of a complex immune cell microenvironment.

## Additional files

Additional file 1: Figure S1. MMTV-PyMT.CCR5^−/−^ mice develop smaller tumors that exhibit reduced glucose uptake compared with MMTV-PyMT.CCR5^+/+^ mice. **A** MMTV-PyMT.CCR5^+/+^ (*n* = 2) and MMTV-PyMT.CCR5^−/−^ mice (n = 2) were followed for tumor development as described in Fig. 1. **B** 18 days post-tumor onset the volume of each individual tumor in each mouse was quantified using a microCT scanner (GE Locus Ultra). **C** Mice were administered 10 MBq of [^18^F]FDG, then after 1 h tumor glucose uptake was measured using a microPET scanner. Standardized uptake value (SUV) of glucose was measured. The value was normalized to tumor volume and blood glucose level. * p < 0.05. (PDF 65 kb)

Additional file 2: Figure S2. Tumors from MMTV-PyMT.CCR5^−/−^ mice have a lower perfusion index than tumors from MMTV-PyMT.CCR5^+/+^ mice. Following microCT and microPET scanning, the same mice that were employed for Additional file 1: Figure S1 were injected iv with 40mgkg^−1^ of Hoechst 33,258, then euthanized 60 s later. Tumors were harvested, frozen in liquid nitrogen and tumors of equivalent size (0.4-0.5 cm diameter, 250-500 mm^3^ volume) were sectioned at 8 μm (**A,B)**. **C** Tumor perfusion was measured using ImageJ after visualizing the sections with UV illumination. The perfusion index was normalized to background. Values are the means ± S.E. of technical triplicates. * p < 0.05. (PDF 394 kb)

Additional file 3: Figure S3. CD45 cells infiltrate both MMTV-PyMT.CCR5^−/−^ and MMTV-PyMT.CCR5^+/+^ tumors. MMTV-PyMT.CCR5^+/+^ (*n* = 3) and MMTV-PyMT.CCR5^−/−^ (n = 3) mice were euthanized 18 days post-tumor onset. Tumors of 0.4-0.5 cm diameter were harvested, sectioned and stained for CD31 (endothelial cells) and CD45 (immune infiltrates). Representative images are presented with and without DAPI (nuclear DNA) staining. (PDF 130 kb)

Additional file 4: Figure S4. Generation of MDA-MB-231.CCR5^−/−^ using CRISPR/Cas9. The knockout cassette carries puromycin resistance. **A** Candidate MDA-MB-231.CCR5^−/−^ cells were first screened for viability in the presence of 1 μg/mL of puromycin. Subsequently, candidate MDA-MB-231.CCR5^−/−^ cell lines were confirmed CCR5 null by **B** PCR and **C** staining with an anti-CCR5 antibody. (PDF 129 kb)

## Abbreviations

2-DG: 2-deoxy-D-Glucose
2-NBDG: 2-(N-(7-Nitrobenz-2-oxa-1,3-diazol-4-yl)Amino)-2-Deoxyglucose
9-AA: 9-Aminoacridine Hydrochloride
AMPK: AMP-activated Protein Kinase
CAFs: Cancer-associated Fibroblasts
CRISPR/Cas9: Clustered Regularly Interspaced Short Palindromic Repeats/Cas9
ER: Estrogen Receptor
GLUT-1: Glucose Transporter-1
H&E: Hematoxylin and Eosin
HER-2: Human Epidermal Growth Factor Receptor 2
HIF-1α: Hypoxia-inducible factor 1-alpha
MALDI-FTICR-MSI: Matrix-Assisted Laser Desorption/Ionization Fourier Transform Ion Cyclotron Resonance Mass Spectrometry Imaging
MDSCs: Myeloid Derived Suppressor Cells
MMTV-PyMT: Mouse Mammary Tumor Virus – Polyomavirus Middle T-antigen
mTOR: Mechanistic Target of Rapamycin
MTT: 3-(4,5-Dimethylthiazol-2-yl)-2,5-Diphenyltetrazolium Bromide
NSG: NOD scid gamma
PR: Progesterone Receptor
TAM: Tumor-associated Macrophages
Tregs: Regulatory T Cells
VEGF: Vascular Endothelial Growth Factor
