# Supplementary material for: CCL5-CCR5 interactions modulate metabolic events during tumor onset to promote tumorigenesis
Source: BMC Cancer. 2017 Dec 8;17:834. doi: 10.1186/s12885-017-3817-0 (PMC5721608; doi:10.1186/s12885-017-3817-0)
Supplement: Supplementary file 1 — MMTV-PyMT.CCR5−/− mice develop smaller tumors that exhibit reduced glucose uptake compared with MMTV-PyMT.CCR5+/+ mice. A MMTV-PyMT.CCR5+/+ (n = 2) and MMTV-PyMT.CCR5−/− mice (n = 2) were followed for tumor development as described in Fig. 1. B 18 days post-tumor onset the volume of each individual tumor in each mouse was quantified using a microCT scanner (GE Locus Ultra). C Mice were administered 10 MBq of [18F]FDG, then after 1 h tumor glucose uptake was measured using a microPET scanner. Standardized uptake value (SUV) of glucose was measured. The value was normalized to tumor volume and blood glucose level. * p < 0.05. (PDF 65 kb) [file 12885_2017_3817_MOESM1_ESM.pdf]

Figure S1

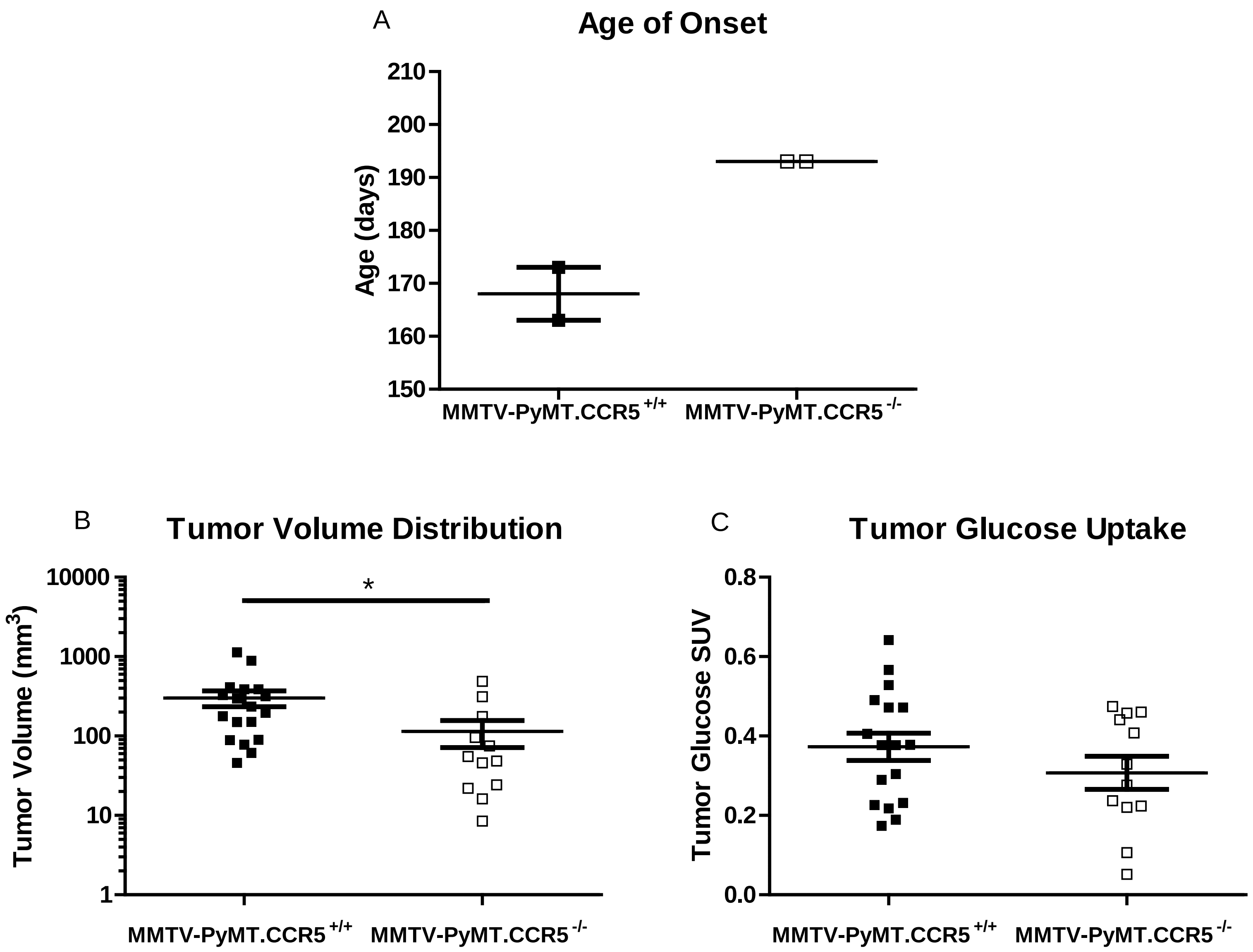
A
Age of Onset
Age (days)
210
200
190
180
170
160
150
MMTV-PyMT.CCR5 +/+
MMTV-PyMT.CCR5 -/-
B
Tumor Volume Distribution
Tumor Volume (mm3)
10000
1000
100
10
1
*
MMTV-PyMT.CCR5 +/+
MMTV-PyMT.CCR5 -/-
C
Tumor Glucose Uptake
Tumor Glucose SUV
0.8
0.6
0.4
0.2
0.0
MMTV-PyMT.CCR5 +/+
MMTV-PyMT.CCR5 -/-
