# Supplementary figures and images for: CCL5-CCR5 interactions modulate metabolic events during tumor onset to promote tumorigenesis
Source: BMC Cancer. 2017 Dec 8;17:834. doi: 10.1186/s12885-017-3817-0 (PMC5721608; doi:10.1186/s12885-017-3817-0)

Figure S2

A

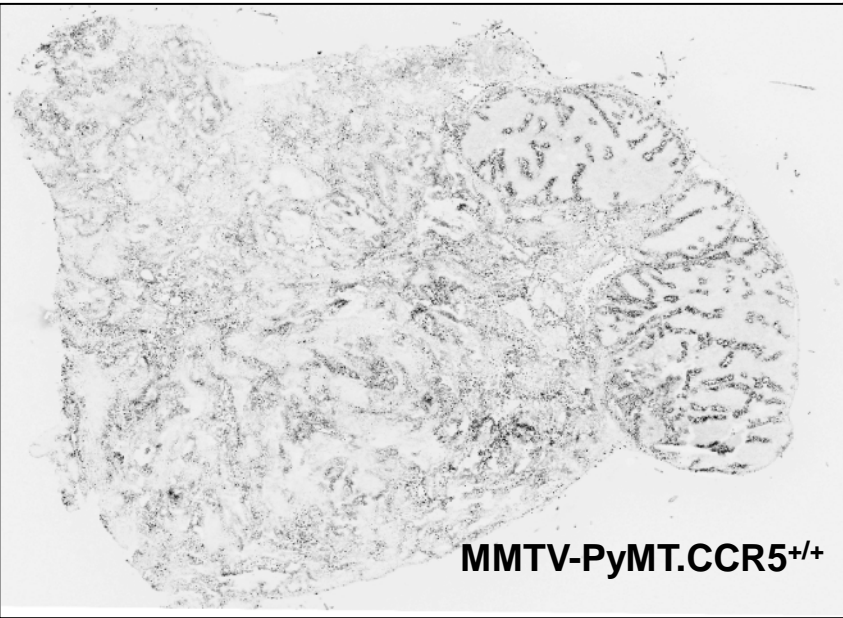

B

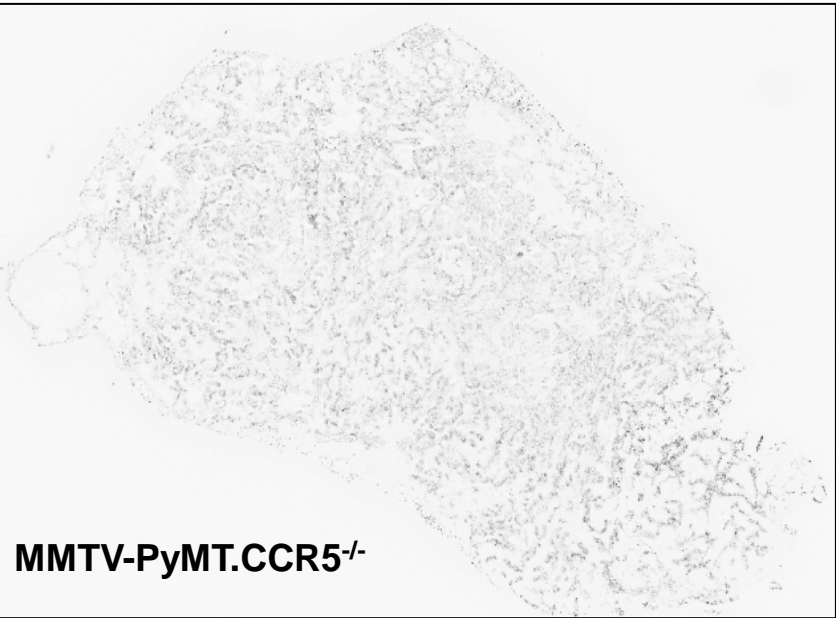

C

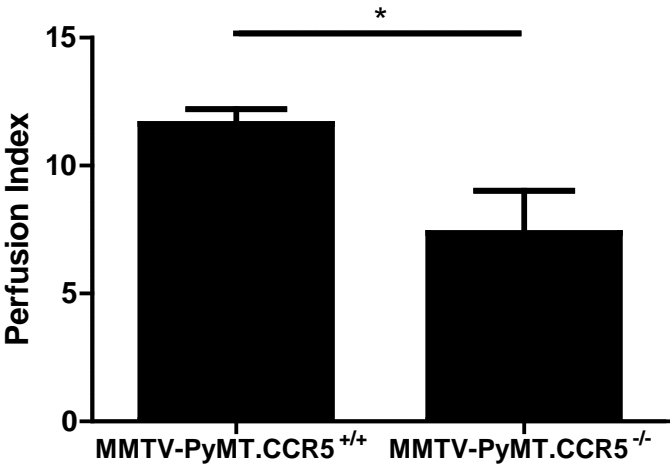

Supplement: Supplementary file 2 — Tumors from MMTV-PyMT.CCR5−/− mice have a lower perfusion index than tumors from MMTV-PyMT.CCR5+/+ mice. Following microCT and microPET scanning, the same mice that were employed for Additional file 1: Figure S1 were injected iv with 40mgkg−1 of Hoechst 33,258, then euthanized 60 s later. Tumors were harvested, frozen in liquid nitrogen and tumors of equivalent size (0.4-0.5 cm diameter, 250-500 mm3 volume) were sectioned at 8 μm (A,B). C Tumor perfusion was measured using ImageJ after visualizing the sections with UV illumination. The perfusion index was normalized to background. Values are the means ± S.E. of technical triplicates. * p < 0.05. (PDF 394 kb) [file 12885_2017_3817_MOESM2_ESM.pdf]

Figure S3

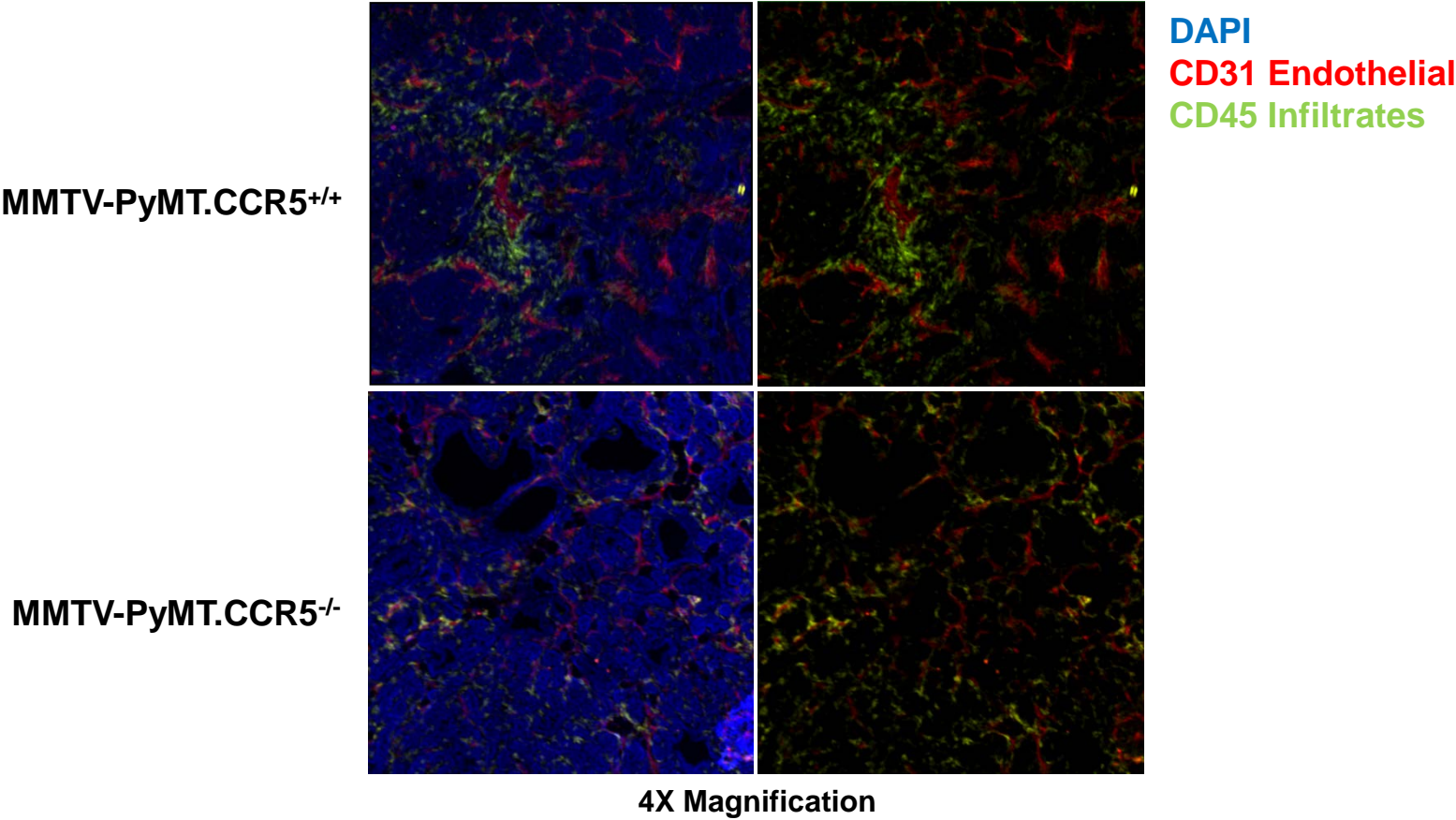

Supplement: Supplementary file 3 — CD45 cells infiltrate both MMTV-PyMT.CCR5−/− and MMTV-PyMT.CCR5+/+ tumors. MMTV-PyMT.CCR5+/+ (n = 3) and MMTV-PyMT.CCR5−/− (n = 3) mice were euthanized 18 days post-tumor onset. Tumors of 0.4-0.5 cm diameter were harvested, sectioned and stained for CD31 (endothelial cells) and CD45 (immune infiltrates). Representative images are presented with and without DAPI (nuclear DNA) staining. (PDF 130 kb) [file 12885_2017_3817_MOESM3_ESM.pdf]

Figure S4

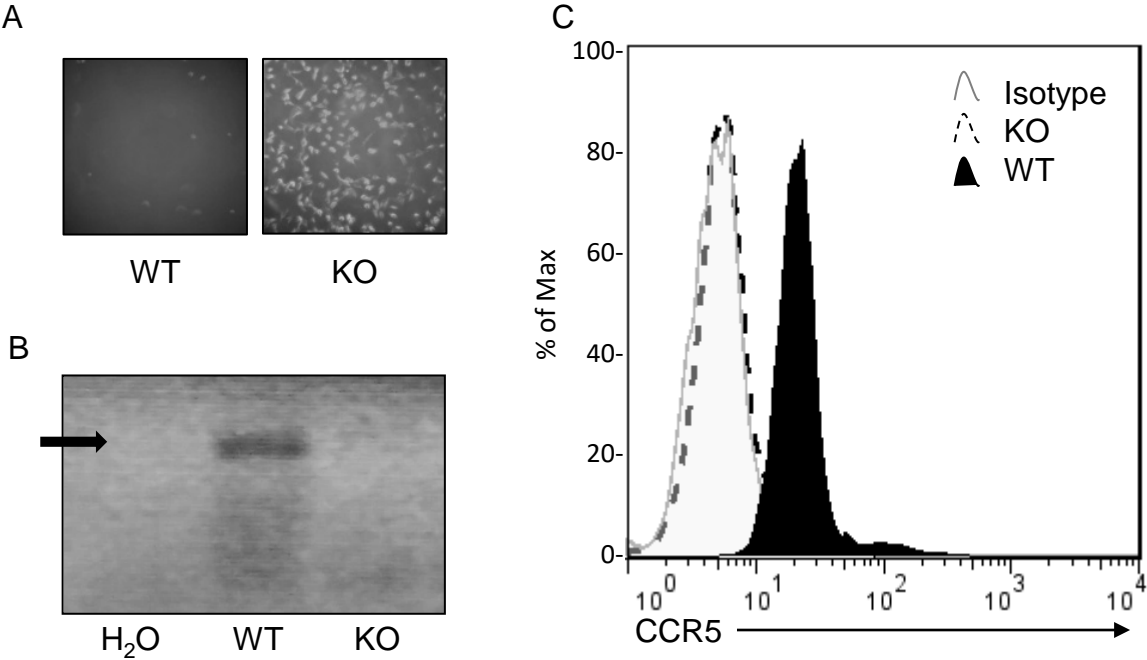

Supplement: Supplementary file 4 — Generation of MDA-MB-231.CCR5−/− using CRISPR/Cas9. The knockout cassette carries puromycin resistance. A Candidate MDA-MB-231.CCR5−/− cells were first screened for viability in the presence of 1 μg/mL of puromycin. Subsequently, candidate MDA-MB-231.CCR5−/− cell lines were confirmed CCR5 null by B PCR and C staining with an anti-CCR5 antibody. (PDF 129 kb) [file 12885_2017_3817_MOESM4_ESM.pdf]
